# Three distinct physical behavior types in fatigued patients with multiple sclerosis

**DOI:** 10.1186/s12984-019-0573-1

**Published:** 2019-08-23

**Authors:** H. E. M. Braakhuis, M. A. M. Berger, G. A. van der Stok, J. van Meeteren, V. de Groot, H. Beckerman, J. B. J. Bussmann, on behalf of the TREFAMS-ACE study group, V. de Groot, H. Beckerman, A. Malekzadeh, L. E. van den Akker, M. Looijmans, S. A. Sanches, J. Dekker, E. H. Collette, B. W. van Oosten, C. E. Teunissen, M. A. Blankenstein, I. C. J. M. Eijssen, M. Rietberg, M. Heine, O. Verschuren, G. Kwakkel, J. M. A. Visser-Meily, I. G. L. van de Port, E. Lindeman, L. J. M. Blikman, J. van Meeteren, J. B. J. Bussmann, H. J. Stam, R. Q. Hintzen, H. G. A. Hacking, E. L. Hoogervorst, S. T. F. M. Frequin, H. Knoop, B. A. de Jong, G. Bleijenberg, F. A. J. de Laat, M. C. Verhulsdonck, E Th L van Munster, C. J. Oosterwijk, G. J. Aarts

**Affiliations:** 1 Department of Rehabilitation Medicine, Erasmus MC University Medical Center, Rotterdam, The Netherlands; 2 Faculty of Health Nutrition and Sport, The Hague University of Applied Sciences, The Hague, The Netherlands; 3 Department of Rehabilitation Medicine, Amsterdam University Medical Centers, Amsterdam, The Netherlands; 4 Amsterdam Public Health Research Institute, Amsterdam University Medical Centers, Amsterdam, The Netherlands; 5 MS Center Amsterdam, Amsterdam, The Netherlands

**Keywords:** Physical behavior, Multiple sclerosis, Principal component analysis, Cluster analysis

## Abstract

**Background:**

Multiple sclerosis often leads to fatigue and changes in physical behavior (PB). Changes in PB are often assumed as a consequence of fatigue, but effects of interventions that aim to reduce fatigue by improving PB are not sufficient. Since the heterogeneous nature of MS related symptoms, levels of PB of fatigued patients at the start of interventions might vary substantially. Better understanding of the variability by identification of PB subtypes in fatigued patients may help to develop more effective personalized rehabilitation programs in the future. This study aimed to identify PB subtypes in fatigued patients with multiple sclerosis based on multidimensional PB outcome measures.

**Methods:**

Baseline accelerometer (Actigraph) data, demographics and clinical characteristics of the TREFAMS-ACE participants (*n* = 212) were used for secondary analysis. All patients were ambulatory and diagnosed with severe fatigue based on a score of ≥35 on the fatigue subscale of the Checklist Individual Strength (CIS20r). Fifteen PB measures were used derived from 7 day measurements with an accelerometer. Principal component analysis was performed to define key outcome measures for PB and two-step cluster analysis was used to identify PB types.

**Results:**

Analysis revealed five key outcome measures: percentage sedentary behavior, total time in prolonged moderate-to-vigorous physical activity, number of sedentary bouts, and two types of change scores between day parts (morning, afternoon and evening). Based on these outcomes three valid PB clusters were derived.

**Conclusions:**

Patients with severe MS-related fatigue show three distinct and homogeneous PB subtypes. These PB subtypes, based on a unique set of PB outcome measures, may offer an opportunity to design more individually-tailored interventions in rehabilitation.

**Trial registration:**

Clinical trial registration no ISRCTN 82353628, ISRCTN 69520623 and ISRCTN 58583714.

**Electronic supplementary material:**

The online version of this article (10.1186/s12984-019-0573-1) contains supplementary material, which is available to authorized users.

## Background

Multiple sclerosis (MS) affects around 2.3 million (young) adults worldwide and leads to changes in the central nervous system that often result in impaired physical and cognitive functions [1–3]. Consequently, the majority of the patients experience fatigue and show different physical behavior (PB) compared to healthy controls [4–6]. In clinical practice, changes in PB are often assumed as a consequence of fatigue, but a number of studies show that PB and MS-related fatigue are only weakly associated [5, 7, 8]. In other words, the role of PB in MS-related fatigue is not straightforward. Several interventions, including exercise training, have been developed to reduce fatigue by improving physical behavior, but the results are insufficient [9]. One explanation for this is that in MS patients, not only the general symptomology is heterogeneous, but also the response to exercise seems highly heterogeneous [10, 11]. As a consequence, considerable variability might be present in the symptoms of fatigue, PB, and in their interaction across and within patients [12, 13]. This suggests that patients with similar levels of fatigue are likely to show varying PB, and that interventions do not match PB starting levels of all patients. More insight in the variability of PB in fatigued MS patients is needed, as better understanding will contribute to the development of more personalized interventions and improve disease management in rehabilitation [10]. To date, the heterogeneity of PB at the start of interventions for fatigued MS patients has not been considered.

To achieve a better and clinically meaningful understanding of the variability of PB in MS rehabilitation, identifying subtypes with comparable PB levels is a suitable approach. A potentially useful method therefore is data-driven clustering based on PB [14], as shown by previous studies in breast cancer patients and in patients with COPD [15]. Using PB as input for identification of subtypes is a challenge, because it is operationalized in several ways in MS [16, 17]. Often, PB is expressed with one outcome measure (e.g. number of steps, or amount of time in a certain activity level). Multiple aspects of PB, however, seem to be affected by MS compared to healthy controls [5], such as the duration and distribution of PB ‘bouts’, with bout defined as a uninterrupted period of a specific type of PB (e.g. sedentary behavior, moderate-to-vigorous physical activity [MVPA]). Only one outcome of physical activity (PA) might be insufficient to evaluate and effectively change a patient’s PB, which makes it reasonable to quantify PB with multiple measures [18]. Assessment should take multiple dimensions such as intensity, type, duration and frequency into account, as well as temporal features, and these characteristics can all be expressed with several potentially relevant measures [12, 16, 19, 20]. Nevertheless, an overkill of measures on PB will limit the clinical interpretation and application, so it should be reduced to a set of measures with minor overlap. Literature shows that this can be realized by statistical data reduction techniques [15].

Combining both data reduction techniques and data-driven clustering enables exploration of the variability of PB in patients based on multiple components of PB. To our knowledge, no study has identified subtypes based on PB in fatigued MS patients, taking the multidimensionality of PB into account. This study therefore aimed to identify subgroups based on PB among fatigued MS patients based on a set of multidimensional PB outcome measures. In addition, potential differences in other patient characteristics between subgroups were assessed.

## Methods

### Participants and data collection

This study used cross-sectional baseline data from the TREFAMS-ACE program [21] for secondary analysis (*n* = 266). TREFAMS is an acronym for the TReating FAtigue in MS program, and ACE refers to the rehabilitation treatment methods under study, i.e. Aerobic training, Cognitive Behavioral Therapy, and Energy Conservation Management. Data were collected from fatigued MS patients who met the following inclusion criteria: i) diagnosed with MS and severe fatigue indicated by a score of ≥35 on the fatigue subscale of the Checklist Individual Strength (CIS20r); ii) ambulatory status (i.e., Expanded Disability Status Scale (EDSS) score < 6); iii) no diagnosis of depression (i.e., Hospital Anxiety and Depression Scale score < 11); iv) no initiation or change to pharmacologic treatment for fatigue during the previous 3 months; and v) aged 18–70 years. The protocol for this study was approved by the Medical Ethics Committee of the VU University Medical Center and informed consent was provided by all participants.

Demographics, body mass index (BMI), type of MS, the disease severity score on the EDSS and fatigue with the CIS20r subscale were collected. The fatigue subscale of the CIS20r includes subjective experience of fatigue in the past 2 week based on eight items scored by a 7-point scale. The score ranges from 8 to 56 with higher scores representing more fatigue [21]. PB was assessed using a 3-dimensional accelerometer (ActiGraph GT3X+ model; 4.6 × 3.3 × 1.5 cm; 19 g) during 7 consecutive days [8]*.* Participants wore the accelerometer around their waist with an elastic belt during waking hours in their daily environment, except during water-related activities. The ActiGraph accelerometer has been proven valid and reliable in patients with MS [22].

### Physical behavior measures

Accelerometer pre-processing was performed as described by Blikman et al. [5]. The accelerometer signals were sampled with a frequency of 30 Hz and analyzed using ActiLife (6.6.2) and MATLAB (R2011b) and the same cut-off boundaries for intensity categories (sedentary, light and MVPA) were used [5]. Accelerometer data had to be available for at least 5 days with a minimum wear time of 660 min. Since PB is approached multidimensional, PB measures were divided into three categories (amount and intensity, frequency and duration, and day patterns). Categories were based on recommendations in literature on operationalization [5, 16, 20, 23]. Each category was divided into two domains, physical activity (PA) and sedentary behavior (SB) [18], which included one or more representative outcome measures calculated by the Actigraph data (Additional file 1).

### Data analysis

#### Principal component analysis

Operationalization of PB measures led to 15 measures in three categories and two domains (Additional file 1), standardized in Z-scores. Principal component analysis (PCA) in SPSS v24.0 was used to reduce the amount of outcome measures. The Kaiser-Meyer-Olkin (KMO) test (KMO value > 0.5) was used to verify whether the 15 measures were suitable for PCA. Before conducting PCA, outlier analysis as recommended by Hair & Black was executed [24]. Single outlier measurements were changed into missing values. PCA was performed using orthogonal direct oblimin rotation since correlations between components were expected due to some overlap between the categories and domains of PB. Selection of the amount of PB outcomes was based on the number of components with eigenvalues ≥1. Number of components was not confirmatory due to the exploratory nature of the analysis. One outcome measure was chosen per component based on high loadings. When multiple outcome measures showed high or comparable loadings, the choice of outcome measure was based on pragmatic reasons to provide a set of measures that is simple to interpret.

#### Cluster analysis

The Z-scores of the PB measures identified in the PCA were uses as input for cluster analysis in SPSS v24.0. Before performing cluster analysis, patients with one or more outlier measurements based on PB were removed. Due to the exploratory nature of the present study and the lack of a priori knowledge of the number of clusters, a two-step combination of a hierarchical and non-hierarchical approach was used [24]. First, agglomerative hierarchical cluster analysis (with squared Euclidian distance) was performed to identify the number of clusters. Decision regarding the number of clusters was based on the rescaled distances in the dendrogram and the percentage of change in agglomeration coefficients at each phase of clustering [24]. Hereafter, a non-hierarchical K-means cluster analysis was performed to improve the initial cluster solution and to minimize the variation within the clusters. Cluster validation was performed by a double-split cross-validation [25]. After splitting the dataset randomly into halves, hierarchical and non-hierarchical cluster analysis was repeated for both datasets. New cluster membership and the cluster centers were saved in an aggregate file. Then, k-means analysis was repeated with the cluster centers of the other random set as input for the next k-means analyses, resulting in two possible cluster solutions per set. Cluster solutions were compared for both sets separately to provide information on sensitivity with Cramer’s V; Cramer’s V closer to one indicates a higher level of agreement [26].

#### Between-cluster differences

Between-cluster differences regarding the demographic and clinical characteristics were evaluated with ANOVA, Kruskall-Wallis and chi-square tests in SPSS v24.0. For the ANOVAs, Bonferroni’s post-hoc test was performed. For the Kruskall-Wallis tests, separate Mann-Whitney U tests were conducted as post-hoc tests. *A p-value* of < *0.05 was considered statistically significant*.

## Results

Table 1 presents demographic and clinical characteristics of participants for whom Actigraph baseline measurements were available for at least 5 days (*n* = 212).

**Table 1:** Characteristics of the study participants (n = 212)

Males/Females	56/156
Age in years, mean (SD)	47.9 (10.4)
Body mass index, mean (SD)	24.1 (4.6)
Type of MS, %	
Relapsing - remitting	155 (73.1%)
Primary progressive	22 (10.4%)
Secondary progressive	21 (9.9%)
Other/unknown	14 (6.6%)
EDSS, median (IQR)	2.5 (1.5)
Duration MS in years, median (IQR)	6.4 (7.5)
Fatigue (CIS20r), mean (SD)	43.8 (7.3)

*EDSS* Expanded Disability Status Scale, *CIS20r* Checklist Individual Strength

A small percentage (0.48%) of all data points, concerning four patients, were considered as outliers and resulted in exclusion. All outlier measurements deviated four to seven times the standard deviation of the mean for several PB measures and were removed [24].

### Principal component analysis

The dataset met the KMO criteria for conducting PCA (KMO = 0.708). PCA identified five key PB components; eigenvalues and explained variance per component are reported in Table 2. Total explained variance was 80.1%. Component 1 was mainly characterized by high loadings on amount and intensity measures, except for total time in sedentary bouts. Components 2 and 5 were characterized by change scores of MVPA and sedentary behavior from morning to afternoon, or afternoon to evening. All high loadings on component 4 were physical activity measures of frequency and duration, whereas high loadings on component 3 were sedentary behavior measures of frequency and duration. The percentage sedentary behavior (%SB), total time (tt) MVPA and sedentary behavior/number of bouts (SB NoB) were chosen as key outcome measures representing the amount and intensity, and the frequency and duration measures. Regarding day pattern measures, %MVPA afternoon minus %MVPA morning (dMVPA1) vs. %SB afternoon minus %SB morning (dSB1), and %MVPA evening minus %MVPA afternoon (dMVPA2) vs. %SB evening minus %SB afternoon (dSB2) showed similar loadings on components. To be consistent in choosing domains, to simplify interpretation we opted for dSB1 and dSB2 since they showed overall highest factor loadings.

**Table 2:** Parameters of physical behavior (i.e. physical activity and sedentary behavior) divided into categories and with their explained variance (%), eigenvalues and loading on the PCA components. For each outcome measure, the highest loading is in bold

			Component				
			1	2	3	4	5
		%Variance (total = 80.1%)	39.59	13.21	10.53	9.07	7.68
		Eigenvalues	5.94	1.98	1.58	1.36	1.15
Amount and intensity	PA	%Active	**0.97**	0.02	0.04	−0.10	−0.04
		%MVPA	**0.78**	0.07	− 0.08	0.36	− 0.10
		CPD	**−0.97**	−0.02	− 0.04	0.10	0.03
		CPM	**0.88**	0.09	0.04	0.20	−0.09
	SB	%SB	**0.92**	0.07	−0.06	0.18	−0.08
Frequency and duration	PA	MPVA BL MVPA NoB	−0.04 − 0.04	0.08 − 0.10	−0.09 0.39	**0.64** **0.48**	0.01 0.04
		tt MPVA	0.42	−0.06	0.04	**0.67**	−0.07
	SB	SB BL	−0.63	0.06	**−0.64**	0.15	−0.03
		SB NoB	−0.16	0.07	**0.94**	−0.01	−0.05
		tt SB	**−0.83**	0.16	0.18	0.16	−0.12
Day pattern	PA	dMVPA1	−0.07	**−0.94**	0.02	0.05	−0.02
		dMVPA2	−0.03	0.21	0.07	−0.21	**0.79**
	SB	dSB1	−0.02	**0.91**	0.05	0.08	0.04
		dSB2	0.00	0.07	0.06	−0.21	**−0.93**

*PA* physical activity, *MVPA* moderate to vigorous activity, *CPD* counts per day, *CPM* counts per minute, *SB* sedentary behavior, *BL* bout length, *NoB* number of bouts, *tt* total time, *dMPVA1* %MPVA afternoon minus %MVPA morning, *dMVPA 2* %MVPA evening minus %MVPA afternoon, *dSB1* %SB afternoon minus %SB morning, *dSB2* %SB evening minus %SB afternoon

### Cluster analysis

**Fig. 1:**
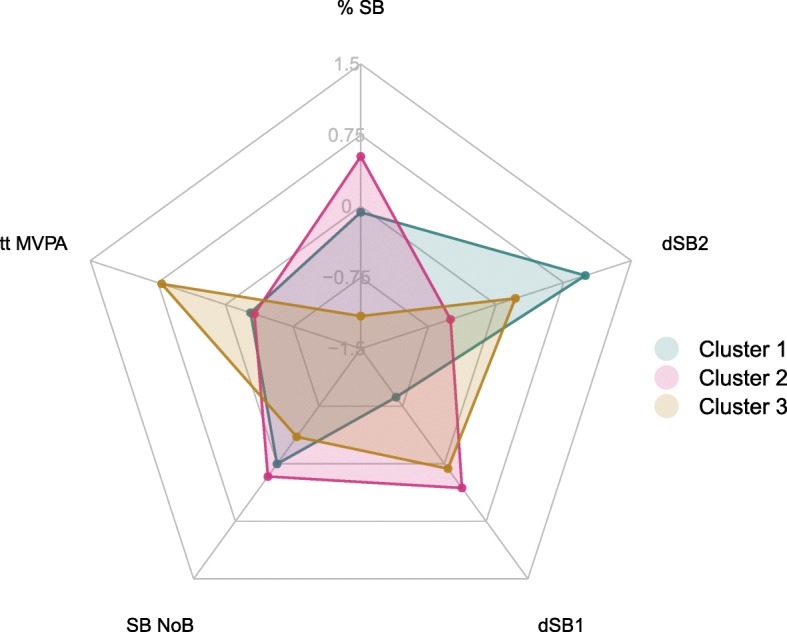
Plot of Z-scores of five key outcome measures of PB per cluster

Agglomerative hierarchical and k-means clustering using %SB, tt MVPA, SB NoB, dSB1 and dSB2 as input parameters resulted in three clusters (cluster 1: *n* = 46, cluster 2: *n* = 114, cluster 3: *n* = 48) as shown by Z-scores in Fig. 1. Cluster 1 can be characterized by a moderate %SB, a low dSB1 value and a high dSB2 value compared to the other clusters. Cluster 2 can be characterized by the highest percentage of SB. Cluster 3 is characterized by the highest value on tt MPVA. SB NoB is comparable for all clusters. Cluster validation was acceptable based on double-split cross-validation (Cramer’s V = 0.7).

### Between-cluster differences

**Fig. 2:**
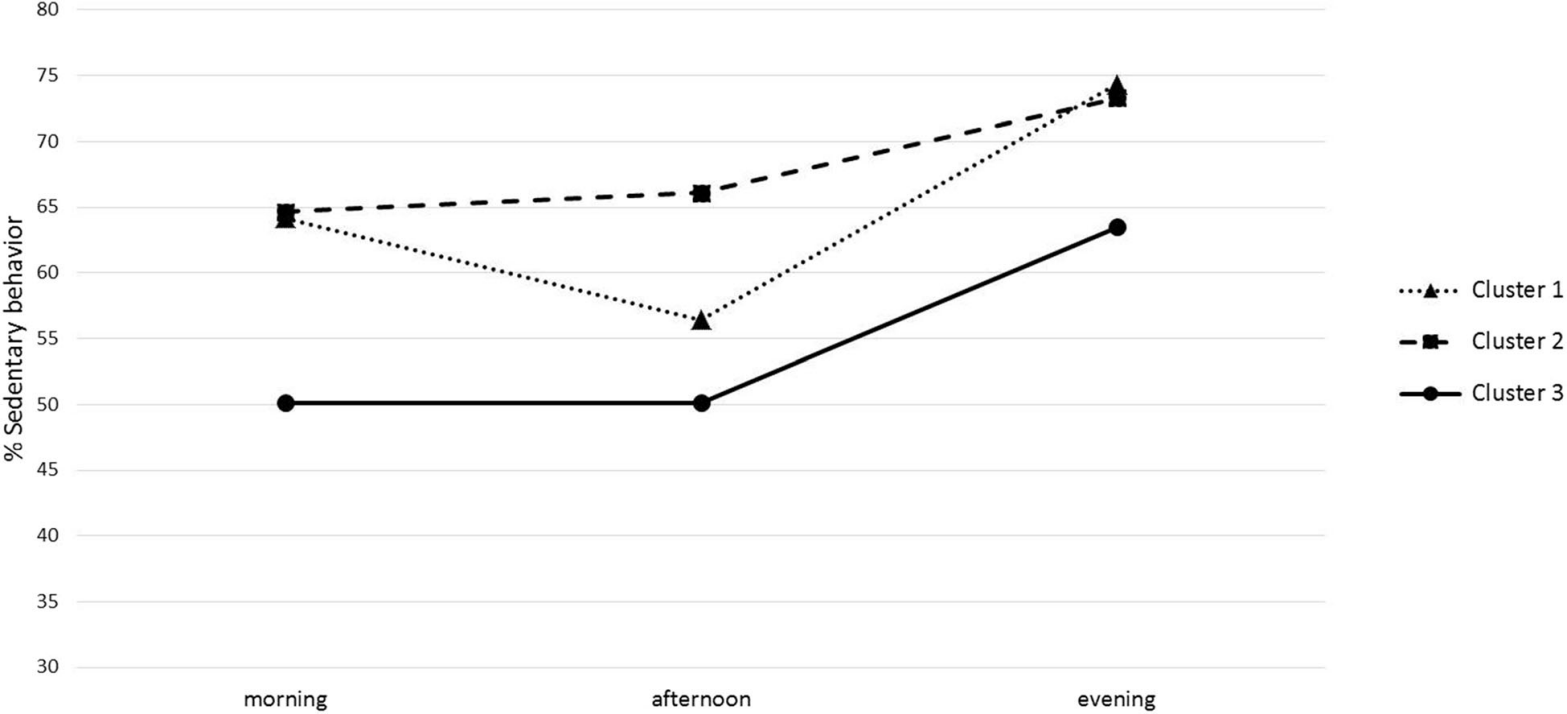
Percentage of sedentary behavior of clusters in the morning, afternoon and evening

The vast majority of PB measures showed significant differences between clusters (Table 3). Figure 2 presents the %SB per day part per cluster and provides insight into differences between dSB1 and dSB2 between clusters. Table 3 shows that dSB1 and dSB2 differ significantly between clusters. Also, Table 3 and Fig. 2 show that cluster 1 is more sedentary in the afternoon compared to the morning (negative dSB1 Z-score), whereas cluster 2 is less sedentary in the afternoon compared to the morning (positive dSB1 Z-score). Cluster 1 and 2 show similar SB in the morning and evening, but cluster 2 is significantly more sedentary in the afternoon. Cluster 3 consisted of the youngest patients (44.4 ± 10.6 years), with age being significantly different compared to cluster 1 (49.8 ± 8.7 years) (*p* = 0.035) (Table 3). EDSS score showed a significant difference between cluster 1 and cluster 3 (*p* < 0.001) and cluster 2 and 3 (p < 0.001). Cluster 3 showed the lowest median EDSS score (2 vs. 3). There were no significant differences in BMI and CIS20r-fatigue scores between the clusters (*p* = 0.166 and *p* = 0.178, respectively).

**Table 3:** Between-cluster differences in patient characteristics and physical behavior measures

	Gender(%male)	TypeMS (% per type)	Age mean ± SD (min-max)	BMI mean ± SD (min-max)	EDSS median (IQR)	Fatigue (CIS20r) mean ± SD (min-max)	Years MS median (IQR)	% SB mean ± SD (min-max)	tt MVPA mean ± SD (min-max)	SB NoB mean ± SD (min-max)	dSB1 mean ± SD (min-max)	dSB2 mean ± SD (min-max)
Cluster 1 (n = 46)	30.4	RR: 80.4 PP: 8.7 SP: 8.7 E/U: 2.1	49.8 ± 8.7 (32.1 – 66.7)	25.1 ± 3.75 (18.5 – 34.8)	3 (1.9)	42.3 ± 7.2(26 - 56)	6.9 (10.3)	63.8 ± 7.5 (50.9 - 82.6)	94.1 ± 67.5 (0.0 - 304.7)	766.6 ± 178.2 (421.0 - 208.0)	-8.8 ± 8.3 (-29.8 - 14.8)	19.5± 6.8 (4.1 - 33.3)
Cluster 2 (n= 114)	27.2	RR:68.1 PP: 11.5 SP: 12.4 E/U: 8.0	46.0 ± 10.7 (19.6 – 66.6)	25.6 ± 5.2 (17.2 – 44.8)	3 (2)	44.2 ± 7.4 (14 – 56)	6.7 (11.9)	69.0 ± 6.2 (56.7 - 84.6)	89.6 ± 62.5 (0.0 - 269.2)	794.7 ± 177.4 (373.0 - 1228.0)	2.1 ± 7.3 (-25.3 - 24.8)	6.8 ± 6.6 (-12.2 -21.5)
Cluster 3 (n = 48)	18.8	RR: 83.3 PP: 6.3 SP: 4.2 E/U: 6.3	44.4 ± 10.6 (24.7 – 68.1)	24.1 ± 3.4 (16.7 – 43.2)	2 (1.5)	44.0 ± 6.6(24 – 54)	4.9 (12.4)	54.2 ± 6.0 (40.5 - 70.7)	195.9 ± 97.8 (41.8 - 452.0)	706.1 ± 135.6 (292.0 - 1003.0)	-0.2 ± 8.6 (-30.4 - 24.5)	12.9 ± 7.3 (-1.7 - 28.2)
*P*	*0.393*	*0.501*	0.032*	*0.166*	<0.001*	*0.178*	*0.389*	<0.001*	<0.001*	0.009*	<0.001*	<0.001*
*Post-hoc test*			*0.111*	*1.000*	*0.454*	*0.067*	*1.000*	<0.001*	*0.809*	*0.391*	<0.001*	<0.001*
			0.035*	*0.839*	<0.001*	*0.168*	*0.510*	<0.001*	<0.001*	*0.118*	<0.001*	<0.001*
			*1.000*	*0.175*	<0.001*	*0.741*	*1.000*	<0.001*	<0.001*	0.002*	*0.063*	<0.001*

*RR* relapsing – remitting, *PP* primary progressive, *SP* secondary progressive, *E/U* else or unknown, *% SB* percentage sedentary behavior, *SB no. bouts* number of bouts in sedentary behavior, *tt MVPA* total time in moderate to vigorous activity, *dSB1* %SB afternoon minus %SB morning, *dSB2* %SB evening minus %SB afternoon

## Discussion

This study aimed to identify subtypes in fatigued MS patients based on multidimensional PB measures. The results show that fatigued MS patients can be categorized in three subtypes with substantial differences in PB. The majority of the patients were classified as cluster 2 and characterized by the highest percentage of sedentary behavior. The most active patients (cluster 3) were characterized by youngest age, and lowest EDSS.

A unique aspect of the cluster analysis was that multiple objective 7-day PB measures in different dimensions specified by PCA were used as input. The main goal of the data reduction by PCA was to avoid an unnecessary number of measures that actually provide similar information and in addition, interpretation of differences between clusters based on fewer outcome measures is preferred. The five components determined by PCA accounted together for 80.1% of the total variance, which is higher than a similar study using PCA in multiple PB measures (60%) [15]. The five components discriminated well but only in the category ‘frequency and duration’ the component loadings differentiated between the domains physical activity and sedentary behavior (Table 2). Component loadings in the categories ‘amount and intensity’ and ‘day pattern’ were more comparable between the domains physical activity and sedentary behavior.

Data-driven cluster analysis yielded three distinct PB subtypes with more homogeneous PB from a heterogeneous sample of fatigued MS patients. The number of patients in each cluster varied. Similar cluster analysis studies also showed an unequal distribution of patients in the clusters [15, 27, 28]. In contrast to similar studies using objective PB measures in other patient populations, we conducted double split cross-validation, which supports performing cluster analysis in this dataset. Even though the number of patients was not equally divided across clusters, results of the validation showed that the sensitivity of our cluster analysis was acceptable.

Comparison of PB between clusters showed that the vast majority of the five key outcome measures showed significant differences (Table 3). Cluster 2 was almost 15% more sedentary based on %SB compared to the most active cluster (cluster 3), meaning that during a day with 16 waking hours, the sedentary patients spent almost 2.5 h in more sitting or lying. Compared to cluster 1, patients in cluster 2 spent around 50 min more in sedentary behavior. However, cluster 1 (SB = 63.8%) and cluster 2 (SB = 69%) patients seem to be slightly less sedentary compared to other chronic neurological conditions, such as stroke (%SB = 74.8%) [29] and Parkinson’s disease (%SB = 75%) [30]. Remarkably, the %SB of cluster 1 showed a significant difference compared to cluster 2, whereas, in contrast, the number of sedentary bouts (SB NoB) was similar. Patients in cluster 2 divided their sedentary behavior into longer uninterrupted bouts and can be seen as more willingly and uninterruptedly sedentary compared to patients in cluster 1. In addition, Fig. 1 shows that both day pattern measures were main causes of the distinction between cluster 1 and 2. In the afternoon, patients in cluster 1 seem to be less sedentary compared to cluster 2, however, they showed similar behavior in the morning and evening (Fig. 2). A possible reason could be that patients in cluster 1, are less engaged in daytime jobs and have more time to be active during the day. Conversely, it is also possible that patients in cluster have more need for an afternoon nap. These findings support earlier studies [20, 31] reporting that the temporal feature of PB is useful to understand patients’ PB. Noteworthy is that dSB1 and dSB2 are relative change scores and they are not completely independent of each other, since both include SB in the afternoon. Nevertheless, component loadings show minor interrelatedness (Table 2). Although challenging, only one easy-to-interpret outcome measure that represents day pattern is recommended in future studies.

In cluster 1 and 2, the minimum of tt MVPA was zero and the standard deviations were relatively high, meaning that several patients did not, or barely met the intensity threshold for MVPA. As a result, a substantial part of these patients did not perform activities with intensities > 3 METs in daily life, such as heavy household activities or sporting activities like brisk walking and cycling. Nevertheless, it can be considered that tt MVPA was the most distinctive measure for cluster 3 compared to the other clusters (Fig. 1). Every patient in cluster 3 met the threshold for at least 41 min per week. Since these active patients even showed slightly less %SB (54.2 ± 6.0%) compared to their healthy peers (57.5 ± 9.4%) [5], it can be concluded that their PB is not affected by MS-related fatigue. In addition, cluster 3 consisted of the youngest patients. Similar results regarding age were found in studies with healthy subjects [32]. In general, older adults are less active than young adults because of e.g. sports and commuting activities [32]. Also other cluster analysis studies showed similar results regarding age [15, 27, 28].

The most important finding was that patients with similar fatigue levels showed large differences in PB. Magnitudes of differences (e.g. 2.5 h more sedentary per day divided into long uninterrupted bouts) can be considered as clinically relevant. Patients who are willingly and mostly uninterruptedly sedentary, like patients in cluster 2, require a different approach compared to patients with similar PB as healthy controls (cluster 3). Other studies support the idea of tailoring intervention approaches, since they showed that sedentary patients are often not willing to change behavior and have low awareness of their personal physical activity levels [33, 34]. In contrast, active patients seem to cope better with their feeling of fatigue since their PA levels are not affected. In other words, fatigue is apparently not a reason to be sedentary for every patient. Likely, motivating patients in cluster 3 to increase their levels of PA even more will not decrease the feeling of fatigue. This supports the thought that the relation between fatigue and PB is not straightforward and as a reason, targeting primarily on PB, even when personalized, will not lead to reduced levels of fatigue for every patient. Still, it is important to maintain a healthy lifestyle including appropriate levels of PA in order to improve other symptoms than fatigue, such as disability, quality of life and incidence of comorbidity [35, 36]. Insight in the PB profile with multiple PB measures therefore has potential as a starting point during counseling sessions to further interrogate the underlying causes of a patients affected PB. Nevertheless, future interventions that target at PB should also consider baseline PB levels since it is highly variable in fatigued MS patients.

### Study limitations

Several limitations of this study need to be addressed. First, since we were restricted to outcome measures that could be calculated from the Actigraph, our selection of PB outcome measures might not be completely comprehensive, we did not measure specific movements or postures like sitting, walking, cycling or running. Comparison with other MS studies is thereby limited since they used other devices and settings [16]. Besides, comparing PB outcomes of different studies and devices should be done with caution, since different operationalization of PB can result in systematic differences in outcomes [37]. Second, since cross-sectional baseline data of the TREFAMS-ACE study were used, no causal associations between PB and fatigue can be drawn. Nevertheless, all participants in this sample ‘approved’ the TREFAMS-ACE interventions and our results support that the PB starting levels were considerably different. Also, the inclusion criterion of severe fatigue was determined with the CIS20r which resulted in no differences in fatigue between clusters. Subsequently fatigue was not heterogeneous in our study sample and generalizability to the total MS population might be limited. Finally, removing outliers from the dataset was rather based on highly exceptional PB and not on technical errors. In four patients, one or more PB measures deviated four to seven times a SD from the mean. In order to maintain generalizability to the fatigued MS population and to successfully conduct our statistical techniques it was decided to exclude four patients.

## Conclusion

This is the first study that explored identification of subtypes based on multidimensional PB in severely fatigued MS patients. Three distinct PB subtypes could be distinguished. The PB subtypes, based on a unique set of PB outcome measures are promising for the design of more individually-tailored PB interventions in rehabilitation. Further research should focus on the clinical feasibility of PB subtypes in the design of interventions.

## Additional file

Additional file 1: Operationalization of physical behavior measures. (DOCX 21 kb)

## Data Availability

The datasets generated and/or analysed during the current study are currently not publicly available due other TREFAMS studies that are still in progress.

## Abbreviations

BMI: Body mass index
CIS20r: Fatigue domain of the Checklist Individual Strength
EDSS: Expanded Disability Status Scale
KMO: Kaiser-Meyer-Olkin
MS: Multiple Sclerosis
MVPA: Moderate-to-vigorous-physical-activity
NoB: Number of bouts
PA: Physical activity
PB: Physical behavior
PCA: Principal component analysis
SB: Sedentary behavior
TREFAMS-ACE: TREating FAtigue in Multiple Sclerosi - Aerobic training, Cognitive Behavioral Therapy and Energy Conservation Management
tt: Total time
