# Supplementary material for: Three distinct physical behavior types in fatigued patients with multiple sclerosis
Source: J Neuroeng Rehabil. 2019 Aug 23;16:105. doi: 10.1186/s12984-019-0573-1 (PMC6708224; doi:10.1186/s12984-019-0573-1)
Supplement: Supplementary file 1 — Operationalization of physical behavior measures. (DOCX 21 kb) [file 12984_2019_573_MOESM1_ESM.docx]

**Additional file 1**

Amount & intensity categories:

- *Mean counts per day (CPD):* the total counts per day based on wear time.
- *Mean counts per minute (CPM):* the mean number of counts per minute per day based on wear time.
- *% active per day (% Active):* percentages of wear time when patients spend time active. ‘Active’ was defined as physical activity (PA) spent above 150 CPM.
- *% MVPA per day (%MVPA):* percentages of wear time when patients spend time moderately to vigorously (MV) PA. MVPA was defined as PA spent equal or above 2691 CPM.
- *% SB per day (%SB):* percentages of wear time when patients spend time in sedentary behavior (SB). SB was defined as SB spent equal or below 150 CPM.

Frequency & duration:
Frequency and duration measures were assessed according to the method of Chastin & Granat (2010). A MVPA bout was defined as at least 1 minute of CPM ≥ 2691. A SB bout was defined as at least 1 minute of CPM below ≤ 150.

- *MVPA bout length (MVPA BL):* mean bout length of MVPA bouts per day.
- *MVPA number of bouts (MVPA NoB):* mean number of MVPA bouts per day.
- *Total time in MVPA bouts (tt MVPA):* total time spent in MVPA more than 1 minute consecutive.
- *SB bout length (SB BL):* mean bout length of SB bouts per day.
- *SB number of bouts (SB NoB):* mean number of SB bouts per day.
- *Total time in SB bouts (tt SB):* total time spent in SB more than 1 minute consecutive.

Day patterns:

Day patterns were analyzed by method of Wolvers et al. (2018). Day pattern parameters represent the change score of percentages MVPA and SB between day parts. Day parts were divided based on time of the day: morning (5:00 AM to 12:00 AM), afternoon (12:00 AM to 6:00PM), and evening (6:00 PM to 12:00 PM).

- *Change score MVPA morning vs. afternoon (dMVPA1):* dMPVA1 = MVPA_afternoon_ - MVPA_morning_
- *Change score MVPA evening vs. afternoon (dMVPA2):* dMVPA2 = MVPA_evening_ – MVPA_afternoon_
- *Change score SB morning vs. afternoon (dSB1):* dSB1 = SB_afternoon_ - SB_morning_
- *Change score SB evening vs. afternoon (dSB2):* dSB2 = SB_evening_ - SB_afternoon_
